# Permeability Behavior of Nanocrystalline Solid Dispersion of Dipyridamole Generated Using NanoCrySP Technology

**DOI:** 10.3390/pharmaceutics10030160

**Published:** 2018-09-17

**Authors:** Ashish Girdhar, Poonam Singh Thakur, Sneha Sheokand, Arvind K. Bansal

**Affiliations:** Department of Pharmaceutics, National Institute of Pharmaceutical Education and Research (NIPER), Sector-67, S. A. S Nagar, Mohali 160062, India; girdharashish1512@gmail.com (A.G.); poonamsinghniper@gmail.com (P.S.T.); snehaniper12@gmail.com (S.S.)

**Keywords:** NanoCrySP, nanocrystalline solid dispersions, permeability, dipyridamole, Caco-2 cells, everted gut sac

## Abstract

Nanocrystals research has been an area of significant interest lately, providing oral bioavailability benefits to solubility- and/or dissolution rate-limited drugs. Drug nanocrystals are generated using top-down or bottom-up technologies. Combination technologies (Nanoedge, Nanopure XP and SmartCrystal) have been recently developed to generate nanocrystals of improved properties. Our lab has also contributed in this field by providing a ‘novel’ platform technology, NanoCrySP, for the generation of nanocrystals. NanoCrySP-generated nanocrystals have improved the oral bioavailability of various molecules. In this study, we aim to assess the permeability behavior of nanocrystals generated by NanoCrySP. Three samples of Dipyridamole (DPM) drug were used in this study: (1) DPM (micron-sized powder), (2) nanocrystals of DPM (NS), generated by media milling (as control) and, (3) nanocrystalline solid dispersion containing DPM (NSD) in the matrix of mannitol (MAN), generated using NanoCrySP technology. In vitro (Caco-2 cell lines) and ex vivo (everted gut sac) studies were conducted in this work. Cellular permeability (*P*_app_) from apical-to-basolateral side in Caco-2 cell monolayer was found to be in the order NS > NSD > DPM, which was the same as their apparent solubility values. Higher *P*_app_ from a basolateral-to-apical side suggested a significant contribution of the P-gp efflux transport for DPM, while NS exhibited much higher inhibition of the efflux mechanism than NSD. Both NS and NSD showed higher permeation from the jejunum region in the ex vivo everted gut sac study. Interestingly, *P*_app_ of NSD was similar to NS in ex vivo everted gut sac model, however, NSD showed higher mucoadhesion than NS and DPM in this study.

## 1. Introduction

A crystalline form of a drug is generally preferred for formulation development over amorphous forms, due to its inherent stability. However, the crystalline form has lesser solubility than its amorphous counterpart which may compromise their oral absorption, especially for Biopharmaceutics Classification System (BCS) class II and IV drugs [1]. The oral bioavailability of such drugs can be limited by either their dissolution or solubility or both [2]. The enhancement of dissolution rate and/or solubility becomes a key factor for the formulation development of such drugs. Nanotechnology interventions such as polymeric nanoparticles, liposomes, solid lipid nanoparticles, and nanocrystals have been widely utilized for this purpose [3,4]. Amongst these approaches, drug nanocrystals have gained interest due to their high drug loading capacity, enhanced drug dissolution and scalability [5].

Nanocrystals can be produced using top-down or bottom-up approaches [5]. Typical top-down techniques are high-pressure homogenization and media milling. Their disadvantages include use of surfactants, long processing times, high energy input, possible contamination from the grinding media and significant downstream processing [6]. Bottom-up processes are primarily precipitation processes and suffer from disadvantages like uncontrolled crystallization and hence wide particle size distribution [7]. Our lab has developed a novel bottom-up spray-drying based technology, NanoCrySP, to produce nanocrystalline solid dispersions wherein drug nanocrystals are embedded in the matrix of a small molecule excipient [8]. Latter also encourages the nucleation of drug crystals. A solution containing drug and excipient is spray dried to obtain powder particles of size 2–50 µm. Each particle consists of drug crystals of size below 1000 nm. The type and concentration of excipient, crystallization properties of the drug and spray-drying process parameters govern the formation of nanocrystals. This technology has been the subject of patents in India, US, and EU [9]. It produces nanocrystalline solid dispersion as a solid powder which can be converted to finished dosage form with minimal downstream processing. Moreover, NanoCrySP-generated nanocrystals fall under ‘Class I’ i.e., biodegradable in nature and size above 100 nm, based on the Nanotoxicological Classification System [10]. This suggests negligible or low toxicity potential by nanocrystals, thus, anticipating the technology as an asset to the field. Therefore, it is imperative to study the biopharmaceutical advantages of this technique over other commercialized techniques for the generation of nanocrystals.

This study investigates the permeability behaviour of nanocrystals generated by NanoCrySP using in vitro and ex vivo tools. Dipyridamole (DPM) was selected as a model drug as it is a BCS class II compound having a log P of 3.95. It is a poorly soluble weak base which displays dissolution-rate limited oral absorption, with an intrincic solubility of 8 µg/mL [11,12]. Additionally, it is autoflourescent in nature due to its aromatic ring system which makes it a good candidate for cell culture experiments [13]. The studies included in this work were (i) in vitro experiments using the Caco-2 cell line bidirectional transport, cellular uptake and absorption–desorption studies, and (ii) ex vivo experiments using everted gut sac (permeability and mucoadhesion studies).

## 2. Materials and Methods

### 2.1. Materials

DPM was received as a gift sample from Dr. Reddy’s Laboratories (Hyderabad, India). Dioctyl sulfosuccinate sodium (DOSS), sodium lauryl sulfate (SLS), lecithin, 4-(2-Hydroxyethyl) piperazine-1-ethanesulfonic acid (HEPES), penicillin-streptomycin-amphotericin solution, ketamine, propranol, furosemide and lucifer yellow were obtained from Sigma-Aldrich (St. Louis, MO, USA). Hydroxy propyl methyl cellulose (HPMC) was purchased from Dow Chemical Company (Midland, MI, USA). Polyvinyl pyrrolidone (PVP K-30) and poloxamer were procured from BASF SE (Ludwigshafen, Germany). Ortho-phosphoric acid, dibasic potassium phosphate and D-Mannitol (MAN) were obtained from S D fine-chem Ltd. (Mumbai, India). 2-(*N*-Morpholino) ethanesulfonic acid hemisodium salt (MES), 3-(4,5-dimethylthiazol-2-yl)-2,5-diphenyltetrazolium bromide (MTT), phosphate buffered saline (PBS), medium 199 and sodium chloride (NaCl) were purchased from Himedia Labs (Mumbai, Maharashtra, India). Dulbecco’s modified eagle medium (DMEM), fetal bovine serum (FBS) and Hank’ balanced salt solution (HBSS) were purchased from Life Technologies Pvt. Ltd. (Bengaluru, India). Non-essential amino acid (NEAA) and trypsin-EDTA solution were procured from GIBCO, Invitrogen corporation (Waltham, MA, USA). Triton-X 100 was purchased from Thermofisher scientific (Waltham, MA, USA). Caco-2 cell lines were received from National Center for Cell Science (NCCS) (Pune, India). HPLC grade methanol was obtained from Rankem Laboratories (Gurugram, India).

### 2.2. Methods

#### 2.2.1. Generation of Nanocrystals of Dipyridamole (DPM)

Nanocrystals of DPM were generated using two different processes- media milling and spray drying for NanoCrySP. Nanocrystals generated using media milling process were taken as positive control for the study. Henceforth the nanocrystals of DPM generated using media milling and spray drying process have been coded as NS and NSD, respectively.

Media milling: A total of 10 mL aqueous solution containing 110 mg HPMC, 70 mg SLS and 20 mg DOSS was prepared in a 15 mL glass vial. 1000 mg of DPM powder was then added in the aqueous solution and dispersed by vortexing for about 2 min. The glass vial containing aqueous dispersion was charged with 5000 mg glass beads of 150–210 µm diameters and a cylindrical magnetic stirring bar of size 10 × 6 mm. The aqueous dispersion was then milled at a stirring speed of 1200 rpm for 24 h. The suspension was transferred to a glass vial, using a pipette and stored at 2–8 °C in a refrigerator until further use.

Spray drying process for NanoCrySP: 400 mg DPM powder was dissolved in 70 mL of methanol and 1600 mg MAN was dissolved in 30 mL of water. The aqueous phase was then slowly added to the organic phase with continuous stirring to prepare a feed solution having 2% *w*/*v* solid content. The feed solution containing DPM and MAN (1:4) was spray dried (LU228 Model, Labultima Ltd., Mumbai, India) using inlet air temperature of 90 °C, atomization pressure of 1.2 Kg /cm^2^, feed flow rate of 4 mL/min and vacuum of 100 mm of water column. These conditions resulted in an outlet temperature of 45 °C. The spray dried powder was stored at 25 °C/0% RH in a vacuum desiccator containing phosphorous pentoxide until further use.

#### 2.2.2. Differential Scanning Calorimetry (DSC)

DSC heating curves were recorded using DSC (Q2000, TA instruments, New Castle, DE, USA) and analysed using Universal Analysis^®^ software, version 4.5A. Samples of 2–3 mg were taken in crimped aluminium pans and subjected to a thermal scan from 25 °C to 185 °C at a heating rate of 20 °C/min. A dry nitrogen purge was maintained at 50 mL/min. The instrument was calibrated using high purity standard of indium prior to analysis.

Heat-cool-heat analysis of DPM was also carried out using DSC. Sample was heated from ambient to 185 °C at a heating rate of 20 °C/min and held isothermally for 1 min. After this, the sample was quenched at the rate of 20 °C/min up to 0°C. In the second heating cycle, sample was heated to 185 °C at a heating rate of 20 °C/min.

#### 2.2.3. Powder X-ray Diffraction (PXRD)

X-ray diffractograms of samples were recorded at room temperature using Bruker’s D8 Advance diffractometer (Mannheim, West Germany) with Cu Kα radiation (1.54 Å), at 40 kV and 40 mA passing through a nickel filter. The analysis was performed in a continuous mode with a step size of 0.01° and a step time of 1s over an angular range of 5−40° 2θ. The diffractograms obtained were analysed with DIFFRAC plus EVA, version 9.0.

#### 2.2.4. Scanning Electron Microscopy (SEM)

The surface morphology of samples was observed under a scanning electron microscope (S-3400, Hitachi Ltd., Tokyo, Japan) operated at an excitation voltage of 25 kV. A double-sided adhesive tape was used on a steel stage for sample mounting. The samples were then sputter coated with gold using ion sputter before analysis.

#### 2.2.5. Dynamic Light Scattering (DLS)

The particle size of DPM in NS and NSD was determined by the DLS technique (Zetasizer NanoZS, Malvern Instruments, Malvern, UK). Briefly, NSD (5 mg) was dispersed in a 10 mL of aqueous medium containing HPMC (100 mg), SLS (100 mg) and DOSS (60 mg) in a 15 mL glass vial. The dispersion was then vortexed for 10 min followed by an ultrasonication (Branson 3510 ultrasonic cleaner, Marshall Scientific, Hampton, NH, USA) for 10 min and analysed. NS was diluted to a concentration of 1 µg/mL using deionized water and then vortexed for 2 min before analysis. Disposable polystyrene cuvette was used for the analysis. Three measurements (ten runs each) were performed with an equilibration time of 10 seconds. A refractive index of 1.67 was used for DPM and the analysis was carried out in backscattering mode at an angle of 173°. The results were reported in terms of *Z*_avg_ (nm), *D*_90_ (nm) and PDI.

#### 2.2.6. Drug Content Determination

DPM content was determined by separately dissolving 10 mg of NSD powder and 10 µL of NS in 1 mL of methanol. The solutions were then vortexed for 2 min and sonicated for 5 min. The samples were filtered using membrane filter of pore size 0.1 µm and after sufficient dilution (1/1000 times), DPM content was determined using HPLC method. The measurements were carried out in triplicate to calculate the mean and standard deviation.

#### 2.2.7. Solubility Study

The apparent solubility of DPM was evaluated in HBSS buffer of pH 6.5 adjusted with MES (referred as apical medium) and pH 7.4 adjusted with HEPES (referred as basolateral medium) and medium 199. An excess amount of DPM powder, NS or NSD powder were separately dispersed into 15 mL of the medium in a conical flask. The mixture was stirred in a mechanical shaking bath at 60 rpm and 37 ± 0.5 ºC, for 120 min. 1 mL of sample was collected at specified time intervals of 15, 30, 60, 90, 120 min and filtered through a nylon membrane syringe filter of pore size 0.1 µm. 100 µL of the filtrate was then diluted with a mixture of orthophosphoric acid (pH 4.6) and methanol (30%:70% *v*/*v*) to a suitable concentration and analysed using HPLC. The solubility data was expressed as an average of three measurements.

#### 2.2.8. HPLC Analysis of DPM

DPM was quantified using an HPLC system consisting of Series 20AD machine (Shimadzu Corporation, Kyoto, Japan). RF-10AXL fluorescence detector (version 3.20, Kyoto, Japan) was used for the analysis of in vitro and ex vivo samples while samples from solubility studies were analysed using a photodiode array detector (SPD-10AP, Shimadzu Corporation, Kyoto, Japan). The stationary phase was Lichrosphere^®^ C18 (200 × 4.6 mm, 5 µm) reversed-phase column and the temperature was maintained at 40 °C. The mobile phase was composed of orthophosphoric acid (pH 4.6) and methanol (30%:70% *v*/*v*) run in an isocratic mode. DPM was eluted through the stationary phase at a flow rate of 1 mL/min at a retention time of 11 min and detected at an excitation wavelength of 285 nm and an emission wavelength of 470 nm.

HPLC method was validated for linearity, repeatability, reproducibility, and accuracy. The linearity was attained over a concentration range of 100–8000 ng/mL with a correlation coefficient of 0.999. The repeatability and reproducibility were confirmed by coefficients of variation of less than 2%.

#### 2.2.9. Cell Culture

Caco-2 cell lines were obtained from NCCS, India at passage no. 49. The cells were grown in DMEM supplemented with 15% FBS, 1% penicillin-streptomycin-amphotericin solution and 1.5% NEAA solution in T-75-cm^2^ tissue culture flasks. The cell cultures were maintained at 37 ºC in a CO_2_ incubator, water jacketed with HEPA Class 100. The incubator had an atmosphere of 95% air/5% CO_2_ and 95% relative humidity (RH). The cells became 80–85% confluent in 10–15 days, estimated by observing and comparing the space occupied by the adherent cells in the flask with that of the unoccupied space under a microscope. The cells were harvested with trypsin-EDTA prior to seeding in culture plates. The Corning^®^ costar filter inserts (0.4 µm pore size, 0.33 cm^2^) were placed in 24-well culture plates and pre-wetted with 100 µL DMEM for 2 min. The cells, equivalent to a density of 7.5 × 10^4^ cells, were pipetted out from the cell suspension and dispensed on the apical side of the filters for seeding. The basolateral side was filled with 800 µL DMEM and the culture plate was kept for incubation of 6 h. The culture medium (i.e., DMEM) was aspirated from both apical and basolateral side after 6h without disturbing the cell monolayer. Fresh culture medium was added to the apical (400 µL) and basolateral (800 µL) side and incubated. The culture medium was changed every alternate day and the procedure was repeated for 29 days. The cells of passage number 57–70 were used for further studies.

#### 2.2.10. Cell Viability Studies

The cells were harvested and seeded in three different 96-well plates at a seeding density of 1.5 × 10^4^ cells per well. DPM solution was prepared in three different concentrations of 25, 500 and 1000 µg/mL using DMEM and methanol (0.5% *v*/*v*). NS and NSD suspensions were prepared in concentrations equivalent to DPM solution using DMEM. The culture plates containing 200 µL of solution or suspensions were incubated for 2, 24 and 48 h. DMEM and methanol, without DPM, were taken as blank. MTT powder was dissolved in PBS to attain a final concentration of 5 mg/mL. 10 µL of this MTT solution was added to each well and incubated for 4 h (37 °C, 5% CO_2_) to allow reduction of a yellow-coloured MTT solution to insoluble purple-coloured formazan crystals. The media was removed by aspirating using a pipette, and the formazan crystals were dissolved in 100 µL of DMSO and incubated for 1 h. Optical density was read at 570 nm and the background was subtracted at 630 nm using SpectraMax^®^ M2/M2 UV spectrophotometer (Molecular Devices, San Jose, CL, USA). The percent cell viability was measured from Equation (1):

(1) $$\mathrm{Cell}\;\mathrm{viability}\;\left(\%\right)=\frac{\mathrm{Signal}-\mathrm{background}}{\mathrm{Blank}-\mathrm{background}}\;\times \;100$$

#### 2.2.11. In Vitro Transport Studies

##### Monolayer Integrity Tests

Cell monolayer integrity was checked by (a) measuring transepithelial electrical resistance (TEER) from one side of the cell monolayer to the other side, (b) using permeability markers and, (c) measuring the amount of a non-transportable fluorescent compound, lucifer yellow, that could have leaked from the apical side to the basolateral side.

The TEER value was measured with a Millipore ERS voltameter (Millipore, Temecula, CA, USA) to evaluate the monolayer integrity. The TEER value was measured by using Equation (2):

(2) $$\mathrm{TEER}=(R_{\mathrm{monolayer}}-R_{\mathrm{blank}})\times A$$

where, *R*_monolayer_ is the resistance of the cell monolayer coupled with the filter membrane, *R*_blank_ is the resistance of the filter membrane and *A* is the surface area of the membrane (0.33 cm^2^ in 24-well plates).

Propranolol (25 µg/mL) and furosemide (25 µg/mL) were utilized as high and low permeability markers, respectively, to evaluate the integrity of Caco-2 monolayer. The amounts of lucifer yellow in the apical and basolateral side were measured at the start, as well as end of the permeability study. An initial stock solution of lucifer yellow (50 mM) was prepared in DMEM and diluted to 100 µM working solution. 250 µL of the working solution was added to the apical side and the basolateral side was filled with 600 µL of basolateral medium. The plate was then kept in a shaker incubator at 37 °C and 60 rpm for 120 min. After 120 min, 150 µL and 300 µL of the samples were withdrawn from the apical and basolateral side, respectively. The samples were analysed by fluorescence spectroscopy at an excitation wavelength of 485 nm and an emission wavelength of 535 nm.

##### Permeability Study

The cell monolayer was washed thrice with PBS (pH 7.4) to remove any traces of DMEM. The apical and basolateral compartments were filled with apical and basolateral medium, respectively, and the plate was kept for incubation for 60 min. Suspensions of DPM, NS and NSD samples (equivalent to 25 µg/mL of DPM) were prepared in apical medium for apical-to-basolateral and in basolateral medium for basolateral-to-apical studies, followed by shaking and sonication for 5 min. For apical-to-basolateral studies, 250 μL of the suspensions of DPM, NS and NSD samples were separately added to the apical side and 600 μL of the basolateral medium was added to the basolateral side. A sample volume of 300 μL was withdrawn from the basolateral side at 15, 30, 60, 90 and 120 min. The withdrawn volume was replaced with fresh pre-warmed (37 °C) basolateral medium each time.

In basolateral-to-apical studies, the suspensions of DPM, NS and NSD (600 μL) were added to the basolateral side, and the 250 μL of the blank apical medium was added to the apical side. Concentration in the apical side was measured by sampling (150 μL) at 15, 30, 60, 90 and 120 min. The plates with inserts were kept on an orbital shaker at 60 rpm in the incubator and the samples were analysed by HPLC as described in Section 2.2.8. The apparent permeability coefficients, *P*_app_ (cm/s), for both apical-to-basolateral and basolateral-to-apical studies were calculated from Equation (3):

(3) $$P_{app}=\left[\frac{dQ}{dt}\right]\times \frac{1}{AC_{0}}$$

where d*Q*/d*t* is the cumulative transport rate (μg/s) defined as the slope obtained by the linear regression of cumulative amount transported and time, *A* is the surface area of the inserts (0.33 cm^2^ in 24-well plate), and *C*_0_ is the initial concentration of the samples on the apical/basolateral side (μg/mL).

The efflux ratio (ER) was calculated from Equation (4) [14,15]:

(4) $$\mathrm{ER}=\frac{P_{app}\;\left(\mathrm{apical}-\mathrm{to}-\mathrm{basolateral}\right)}{P_{app}\;\left(\mathrm{basolateral}-\mathrm{to}-\mathrm{apical}\right)}$$

#### 2.2.12. Cellular Uptake Studies

The Caco-2 cells were seeded at a density of 1.5 × 10^5^ cells per well in a 24-well plate and grown to form a monolayer by incubating at 37 °C and 95% air/5% CO_2_ for 15 days. DMEM was regularly changed after 2–3 days as explained in Section 2.2.9. Suspensions of DPM, NS and NSD were prepared in apical medium at concentrations equivalent to 25 and 50 µg/mL of DPM. The cell monolayer was incubated with suspensions of DPM, NS and NSD for 60 min. The cells were rinsed thrice with PBS (pH 7.4) after 60 min incubation. Time-dependent cellular uptake (30 and 60 min) of all three samples was studied qualitatively using confocal laser scanning microscope (CLSM) (Olympus Fluoview 1000, Olympus, Japan) at 405 nm excitation and 488 nm emission wavelengths.

#### 2.2.13. Absorption and Desorption Studies

The cell suspension in DMEM was taken at a cell density of 75,000 cells/400 µL. Suspensions of DPM, NS and NSD (equivalent to 25 µg/mL) in DMEM were added to the cell suspension and incubated for 120 min. The cell suspension containing samples was then centrifuged at 10,000 rpm for 15 min and the supernatant was removed. The pellet was washed with PBS and 1% *w*/*v* aqueous solution of Triton-X 100 (100 µL) was added to rupture the cells. After 120 min, methanol (200 µL) was added to extract the DPM accumulated in the cells and analysed by HPLC. For desorption studies, DPM-loaded cells were centrifuged after an incubation period of 120 min. The pellet was washed and re-suspended in PBS for 120 min. The cell suspension was again centrifuged at 10,000 rpm for 15 min and the supernatant was withdrawn and analysed by HPLC.

#### 2.2.14. Ex Vivo Study using Everted Gut Sac

##### Permeability Study

The study was conducted with prior permission from Institutional Animal Ethics Committee (IAEC), NIPER (Approval no. IAEC/17/14, approved on 10 January 2017). Male Sprague–Dawley rats (250–270 g) were kept on overnight fasting prior to experimentation with free access to water. The animals were anesthetized using ketamine and then sacrificed. The abdomen was opened by a midline incision and the intestine was cut into parts by keeping the duodenum segment (10 cm from the stomach) and removing the jejunum (15 cm after duodenum) and ileum segment (25 cm from jejunum) for further use. The jejunum and ileum segments were rinsed with a solution of 0.9% *w*/*v* NaCl. The freshly excised intestinal segments were then gently everted on glass rods to expose the mucosa on the outer side. The intestine was tied with silk thread at one end and was filled with medium 199 at 37 °C. The filled intestine was divided into sacs of approximately 4 cm in length, with silk thread and dissected into the pieces. Each sac was then placed in a 50 mL conical flask containing DPM, NS and NSD suspended in 15 mL pre-aerated medium 199 at 37 °C at a concentration equivalent to 25 µg/mL of DPM. The sacs were incubated at 37 °C in a shaker water bath at 60 rpm. The sacs were removed from the medium after 120 min and blotted dry. The sacs were cut open and the serosal fluid was collected in microcentrifuge tubes. Each sac was weighed before and after collection of the serosal fluid to accurately calculate the volume of medium present inside the sac. Length and diameter of sacs were also measured to determine the area. The experiment was performed in triplicate. The *P*_app_ of DPM was calculated by using Equation (5):

(5) $$P_{\mathrm{app}}=\left[\frac{V}{A\times T}\right]\times \frac{C_{1}}{C_{0}}$$

where *V* is the volume (mL) of serosal content, *A* is the area (cm^2^) of intestinal sac, *T* is the time (s) of incubation, *C*_0_ is the initial concentration on mucosal side (ng/mL), while *C*_1_ is the concentration (ng/mL) of the compound on serosal side after 120 min (or 7200 s).

In addition to the permeability study, an intestinal tissue uptake study was also performed to calculate the amount of nanocrystals embedded in the tissue. The intestinal sacs, which were cut open to remove the serosal fluid (as described in the above paragraph), were homogenized in 5 mL PBS (pH 7.4). Methanol (5 mL) was added to the tissue homogenate and the mixture was again homogenized for 5 min followed by centrifugation at 10,000 rpm and 4 ºC, for 20 min. Acetonitrile (10 mL) was added to the mixture followed by shaking for 1 min and centrifugation at 10,000 rpm and 4 ºC, for 20 min. 1 mL of clear supernatant was pipetted out and 50 µL of diazepam solution (1 mg/mL in methanol) was added as an internal standard. The samples were kept shaking for 2 min followed by centrifugation at 15,000 rpm for 15 min and then analysed using HPLC method.

##### Mucoadhesion Study

The extent of penetration of DPM in mucosa and serosa for DPM, NS and NSD suspensions was determined using CLSM. Thin transverse sections of the gut sacs (~35 µm) were cut using a cryomicrotome at −30 ºC after fixing the tissue at an optimal cutting temperature and placed on a glass slide. The slides were analysed at various depth levels in the direction of *Z*-axis under a lens of 10× magnification and at an excitation wavelength of 405 nm and an emission wavelength of 488 nm.

#### 2.2.15. Statistical Analysis

Statistical significance of *P*_app_ values was compared using the paired *t*-test assuming equal variances (Microsoft Excel 2007). The test was considered to be statistically significant if *p* < 0.05.

## 3. Results

### 3.1. Solid-State Characterization

**Figure 1:**
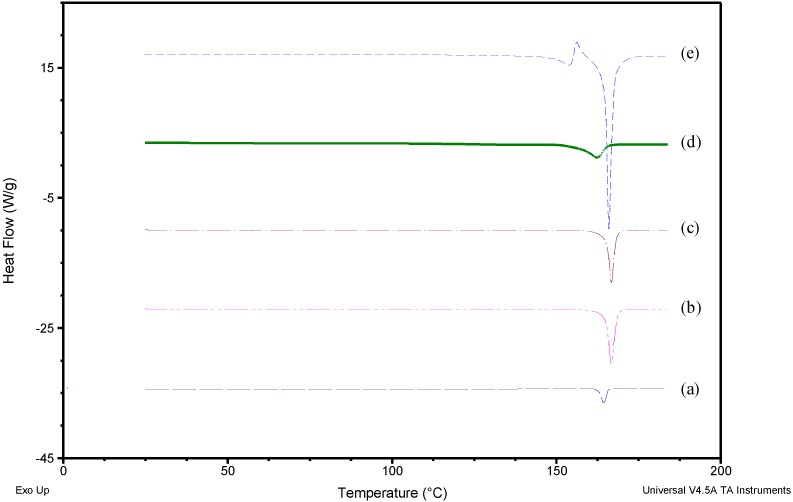
Differential Scanning Calorimeter (DSC) heating curves of (**a**) Dipyridamole (DPM) (**b**) Mannitol (MAN) (**c**) DPM-MAN physical mixture (PM) (**d**) Nanosuspension (NS) and (**e**) Nanocrystalline solid dispersion (NSD).

**Figure 2:**
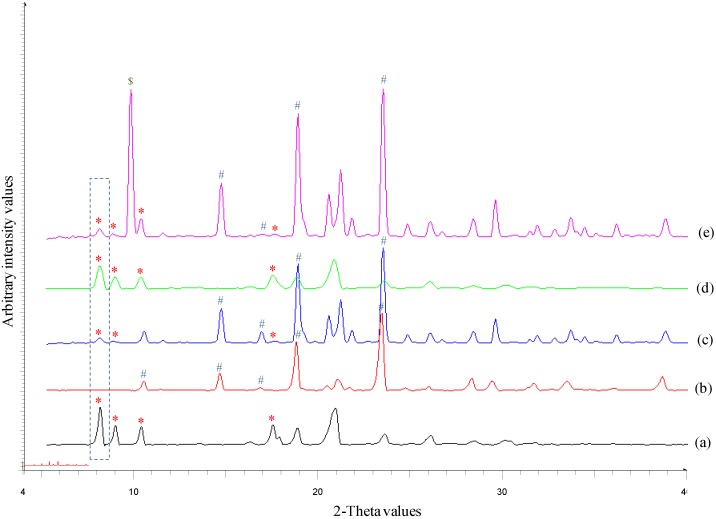
An overlay of X-ray diffractograms of (**a**) DPM (**b**) MAN (**c**) DPM-MAN PM (**d**) NS and (**e**) NSD. The characteristic peaks have been marked with symbols *, # and $ for DPM, β-form of MAN and δ-form of MAN, respectively. The characteristic peak of DPM at 8.1° 2θ value has been marked with a dotted box.

Figure 1 shows the heating curves for DPM, MAN, physical mixture (PM) of DPM and MAN (1:4), NS and NSD. DPM showed a melting endotherm (*T*_m onset_) at 163.04 ºC (51.24 J/g) whereas MAN showed the *T*_m onset_ at 164.94 ºC (281.50 J/g). The melting endotherm of both DPM and MAN in PM appeared at nearly the same temperature (164.76 ºC) and hence, could not be separated out in DSC. Heat-cool-heat analysis provided the glass transition temperature (*T*_g onset_) of DPM at 41.97 ºC (0.5245 J/(g·°C)) and crystallization temperature (*T*_c onset_) at 133.82 ºC (48.65 J/g) (data not shown). NS showed the *T*_m onset_ at 156.79 ºC (38.45 J/g) while NSD showed first melting event at 148.33 ºC (27.03 J/g) corresponding to δ–form of MAN followed by its recrystallization and then a second melting event at 164.86 ºC (195.00 J/g) corresponding to overlapped melting of both DPM and β-form of MAN. Therefore, the melting event of DPM could not be confirmed by DSC. However, the PXRD patterns clearly revealed the major DPM peak at 8.1° 2θ and other smaller DPM peaks (8.8°, 10.1° and 17.4° 2θ) in NS and NSD samples. This indicates that the obtained nanocrystals (in NS and NSD samples) consisted of crystalline DPM (Figure 2). β-form of MAN gives characteristic peaks at 10.2°, 14.8°, 17.1°, 19.1° and 23.5° 2θ values while δ–form of MAN shows its characteristic peak at 9.8° and absence of peaks in the region 10.0–19.0° 2θ values [16]. A mixture of polymorphic forms i.e., β and δ, of MAN was observed in case of NSD (Figure 2e) as is evident from the presence of peaks (10.2°, 14.8°, 17.1°, 19.1° and 23.5° 2θ) of β-form and an intense peak at 9.8° 2θ, corresponding to δ–form.

**Figure 3:**
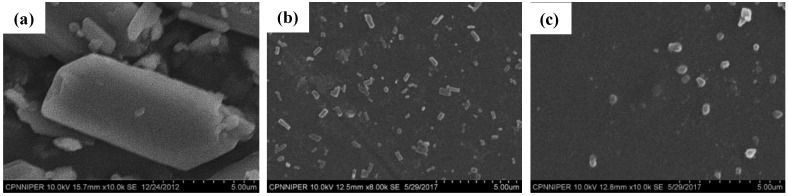
Scanning Electron Microscopy (SEM) photographs of (**a**) DPM, (**b**) NS and (**c**) NSD.

The *Z*_avg_ and *D*_90_ of DPM in NS sample were found to be 373.40 (± 38.20) nm and 260.30 (± 34.90) nm, respectively, with a PDI of 0.10 (± 0.01). NSD showed *Z*_avg_ of 1131.00 (± 79.30) nm and *D*_90_ of 720.00 (± 56.90) nm with a PDI of 0.50 (± 0.20). SEM images (Figure 3) also showed DPM particles of below 1.00 µm in both NS and NSD samples. The DPM contents in NS and NSD sample were found to be 97.39 (±2.55) % and 96.86 (±3.28) %, respectively. No significant variations (*p* > 0.05) between particle sizes of NS and NSD samples were found on storage stability testing for 30 days (data shown in Table S1).

### 3.2. Solubility Study

Solubility studies of DPM, NS, and NSD were performed in three different media i.e., HBSS (pH 6.5), HBSS (pH 7.4) and medium 199. The former two media were used in in vitro studies while the latter was used in ex vivo studies. The order of solubility in all the three media was found to be NS > NSD > DPM (Table 1) at all time points (except at 15 min for medium 199).

### 3.3. Cell Viability Studies

**Figure 4:**
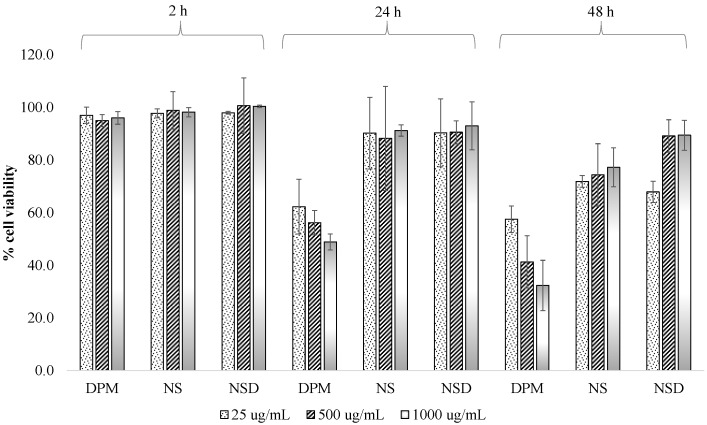
Cell viability (%) of DPM, NS and NSD at different concentrations in Caco-2 cells at three incubation times 2 h, 24 h and 48 h. Values are represented as a mean ± standard deviation.

The viability of Caco-2 cells was measured using the MTT test to evaluate the cytotoxicity of DPM, NS and NSD samples. MTT gives a dark purple formazan product on oxidation by mitochondrial dehydrogenase in living cells. However, mitochondrial dehydrogenase becomes inactive in damaged or dead cells. Figure 4 shows the cell viability of samples at different concentrations and incubation time intervals. Cell viability of >80% ensured that DPM concentrations of 25, 500 and 1000 µg/mL were non-toxic to the cells up to 2 h. After 24 and 48 h, a cell viability of <60% was observed for all three concentrations of DPM. Statistical analysis of the data, using ANOVA with post-hoc Tukey’s test, suggested that the variations in % cell viability of DPM, NS and NSD were insignificant (*p* > 0.05) at all three concentrations, up to 2h. However, significant variations (*p* < 0.05) in % cell viability were observed in DPM vs NS and DPM vs NSD at all three concentrations up to 24 and 48 h. NS and NSD showed no statistically significant variations (*p* > 0.05) between them up to 24 and 48 h, with respect to the concentrations.

### 3.4. In Vitro Transport Studies

TEER values above 300 cm^2^ assured the integrity of Caco-2 cell monolayers formed with tight junctions to allow the passage of samples by transcellular route. *P*_app_ values of the permeability markers, propranolol and furosemide, were found to be 30.40 (±1.23) × 10^−6^ cm/s and 3.21 (±0.27) × 10^−6^ cm/s, respectively. These *P*_app_ values were comparable to the values reported in the literature [17,18,19]. <2% permeation of lucifer yellow across the cell monolayer also complemented the TEER results. *P*_app_ of DPM, NS and NSD samples were then determined using Caco-2 cell monolayers. DPM showed a *P*_app_ of 16.84 (±0.37) ×10^−6^ cm/s at 25 µg/mL which matched its literature reported value [20]. Table 2 shows the *P*_app_ and ER values of all three samples from apical-to-basolateral and basolateral-to-apical. *P*_app_ (basolateral-to-apical) of all the samples was significantly (*p* < 0.01) higher than the *P*_app_ (apical-to-basolateral), which indicated the involvement of an efflux mechanism as reported by P-gp transporters for DPM [20]. NS showed a significant reduction in the ER of DPM by 7.8-fold while NSD showed a 4.2-fold decrease in the ER, as compared to DPM. Reduction in the efflux transport by NS and NSD samples was due to their saturation effect on the transporters [20].

#### 3.4.1. Cellular Uptake Studies

**Figure 5:**
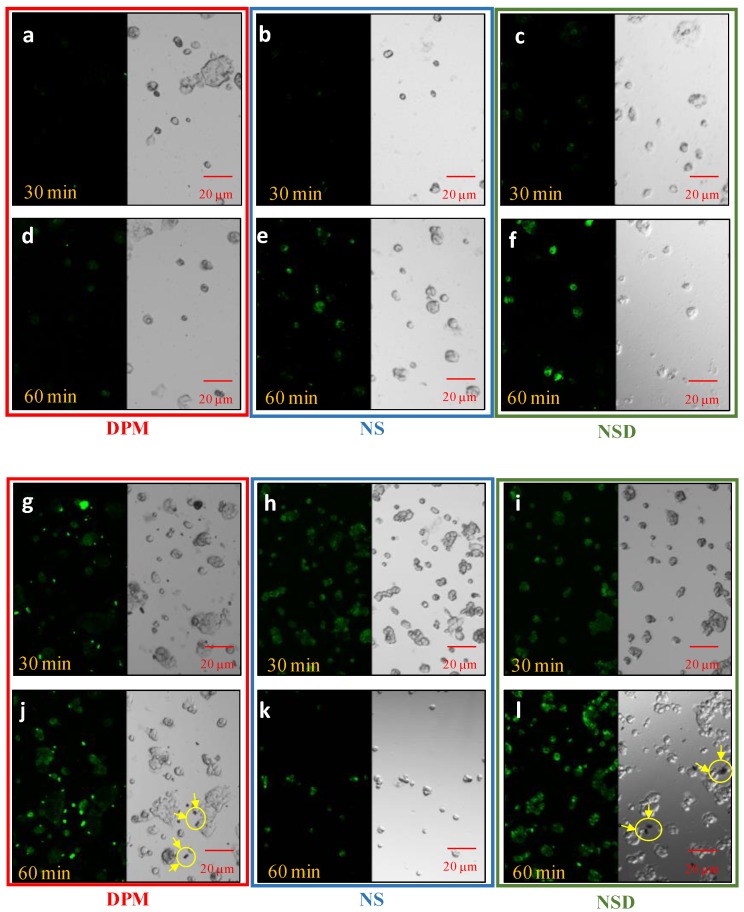
CLSM images of Caco-2 cells after incubation with DPM, NS, and NSD at 25 µg/mL (image **a**–**f**) and 50 µg/mL (image **g**–**l**), respectively for 30 and 60 min. Left side shows the fluorescence of DPM in the laser beam and the right side shows the phase-contrast images. Images (**a**,**d**,**g**,**j**) represent DPM; images (**b**,**e**,**h**,**k**) represent NS and; images (**c**,**f**,**i**,**l**) represent NSD. Encircled zones with arrows indicate particles/agglomerates observed outside the cells.

CLSM was used to analyse the retention of DPM in Caco-2 cell monolayers in DPM, NS, and NSD after 30 and 60 min. As can be seen in Figure 5, differences in the fluorescent intensity between DPM, NS and NSD were found at two different concentrations (25 and 50 µg/mL) and two different time points (30 and 60 min). Negligible uptake was observed in DPM at 25 µg/mL in 30 min and 60 min, and showed presence of undissolved crystals at 50 µg/mL (refer Figure 5j). The fluorescent intensity increased with time and concentration of DPM (in all 3 samples) and reached a maximum at 60 min and 50 µg/mL, respectively. NS and NSD showed higher intensity than DPM, however, Undissolved crystals and agglomerates were observed in NSD sample at 50 µg/mL, 60 min (Figure 5l).

#### 3.4.2. Absorption and Desorption Studies

Absorption is the amount of DPM accumulated inside the cells while desorption is the amount coming out from the cells after suspending them in fresh PBS. Table 3 shows the percentage of DPM absorbed and desorbed from DPM, NS and NSD samples. The absorbed amount of DPM was calculated by considering the amount of initial stock solution as 100% whereas the desorbed amount was calculated by considering the absorbed amount as 100%. The amount of DPM absorbed after an incubation of 120 min was in the order: NS > NSD > DPM. The trend for absorption was similar to that observed for *P*_app_ (apical-to-basolateral) and solubility. The order for desorption was NS = NSD > DPM.

### 3.5. Ex Vivo Study

#### 3.5.1. Permeability Study

Table 4 shows the *P*_app_ values for DPM, NS, and NSD sample in the everted gut sac. The permeability was determined from the jejunum and ileum segments. *P*_app_ was higher from the jejunum segment and followed the order as NS = NSD > DPM where NS and NSD showed similar permeability (*p* > 0.05). *P*_app_ from the ileum segment and intestinal tissue uptake of DPM in jejunal segment were followed the similar order.

#### 3.5.2. Mucoadhesion Study

**Figure 6:**
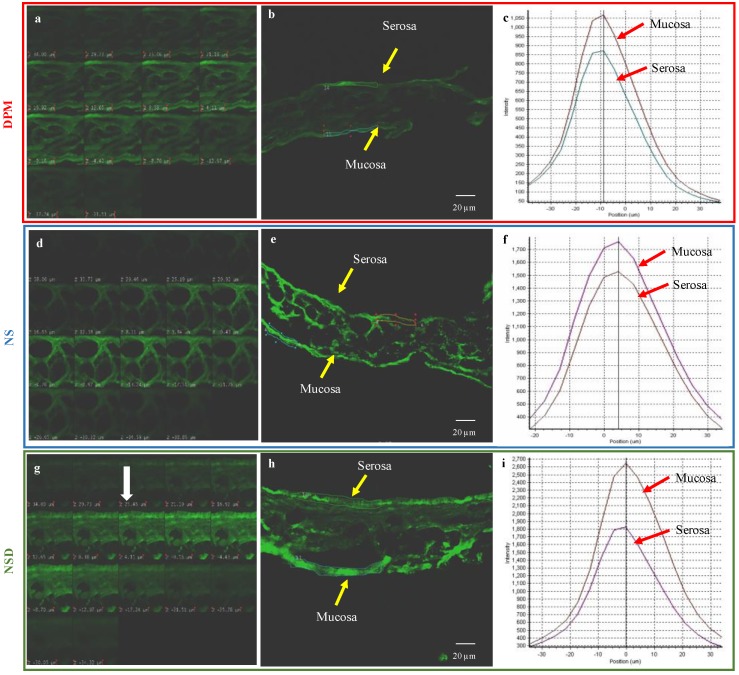
CLSM images of a transverse section of the jejunum segment of the everted gut sac. Images (**a**–**c**) represen DPM, images (**d**–**f**) represent NS and images (**g**–**i**) represent NSD. Images (**a**,**d**,**g**) depict the Z-stack of the sac in increasing depth. Images (**b**,**e**), and h represent the selected images of optimal intensity. Images (**c**,**f**,**i**) represent the measured intensity at serosal and mucosal side.

CLSM images of a 35 µm thick transverse section of the intestinal tissue, showing the fluorescent intensity in *Z*-plane, are as shown in Figure 6. A layer of optimum depth of intensity was selected during the *Z*-stacking. The fluorescent intensity of DPM, NS and NSD was measured in both serosal and mucosal sides of the intestinal tissue. The intensity was then correlated with the amount of DPM. As can be seen from Figure 6, DPM showed an intensity of 850 units in the serosal side and 1050 units in the mucosal side. Similarly, an intensity of 1500 units and 1700 units was observed for NS from the serosal and mucosal sides, respectively. NSD showed an intensity of 1700 units in the serosal side and 2600 units in the mucosal side. These intensity differences between the serosal and mucosal sides for DPM, NS and NSD samples, when compared, pointed towards the higher mucoadhesion tendency of NSD than others. In case of DPM, the large-size crystals could not adhere to the mucous and, therefore, showed no significant intensity difference between mucosal and serosal side.

**Figure 7:**
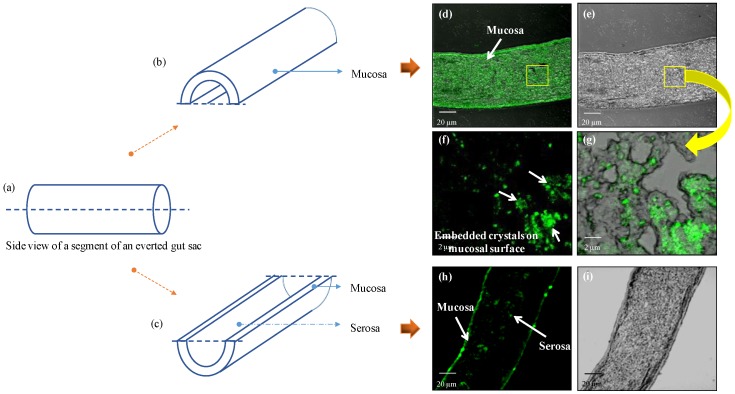
Schematic representation of the mucosal and serosal side on everted gut sac: (**a**) Side view of the jejunum segment of the everted gut sac; (**b**,**c**) depict the mucosal and serosal side of the gut sac after sectioning (longitudinal). CLSM images (**d**–**i**) of NSD treated gut represent: (**d**) phase contrast and (**e**) bright field of the mucosal side. Images (**f**,**g**) represent a zoomed in picture of the images (**d**,**e**) which show the presence of crystals embedded on the mucosal surface. Images (**h**,**i**) represent the phase contrast and bright field of the serosal side of the gut.

In order to further confirm the mucoadhesion of nanocrystals in the intestinal tissue, longitudinal sections of the jejunum segment (treated with NSD) were observed in CLSM. As shown in Figure 7, clear differences in the fluorescence pattern were observed in mucosal and serosal sides. Tiny particles/crystals of NSD, embedded on the mucosal side of the intestinal segment, could be observed. Therefore, NSD sample showed higher mucoadhesion than NS and DPM as was evident from the microscopic analysis (in Figure 6).

## 4. Discussion

According to the Noyes-Whitney equation [21], nanocrystals are well known to increase the dissolution rate of a drug due to decrease in particle size and corresponding increase in the surface area of the drug particles. Besides dissolution rate, particle size reduction has effect on the apparent solubility of the drug and according to Ostwald–Freundlich equation, the apparent solubility increases significantly on reducing particle size below 1000 nm [5]. This is because the reduction of size below 1000 nm increases solvation pressure, fostering an increase in the solubility and also causing disruption of solute-solute interaction which eases the solubilization process [5,22]. Permeability has been reported to be modulated by (a) concentration gradient (b) particle size of the formulation (c) alteration of cell membrane integrity and (d) inhibition of efflux transporters [23,24,25]. In the next few sections, we will discuss the potential reasons for increase in the permeability of DPM in nanocrystals in comparison to their micronized counterpart.

The sequence of *P*_app_ through Caco-2 cells (apical-to-basolateral) was NS > NSD > DPM, and NS = NSD > DPM through everted gut sac (mucosa-to-serosa). The samples for permeability studies were suspensions containing different solubilised portions of DPM. The solubilized portion for DPM, NS and NSD were 5.10 (±0.90), 6.32 (±0.82) and 6.07 (±1.20) µg/mL, respectively, in HBSS (pH 6.5). The time-dependent solubility data in this medium is shown in Table 1. It is evident that the ‘difference in solubility’ increased in a time-dependent manner. The solubility difference for NS and NSD w.r.t. DPM was 1.64- and 1.44-fold, respectively. Possibly, the ‘difference in permeability’ were contributed by the concentration-gradient due to increased solubilisation as well as dissolution efficiency. These two simultaneously occurring phenomenon took place more efficiently in case of NS as it provided discrete particles suspended in the medium against agglomerates seen in NSD (Figure 5j,l). *P*_app_ (basolateral-to-apical) was also evaluated in Caco-2 cells wherein the sequence observed was DPM > NSD > NS. The possible reason for this could be saturation of the transport system because the concentration-dependent inhibition of the P-gp efflux has already been reported for DPM in the literature [20].

Nanocrystals have been reported to be internalized by the cells in a size-dependent manner. Previous reports have indicated efficient internalization of particles less than 200 nm [24]. The results of cell internalization (or absorption-desorption) studies established increased internalization in case of NS than NSD. Though the initial particle sizes (*D*_90_) for NS and NSD were 260.3 (±34.9) nm and 720.0 (±56.9) nm, solubilisation in the buffer medium could have decreased the size of the crystals in a time-dependent manner (as discussed in the above paragraph). This would have contributed to cellular internalization over a period of time. It is also reported that presence of surfactants contributes to enhanced internalization due to its impact on the biochemistry of the cell membrane [26,27]. Therefore, NS showed enhanced internalization due to the presence of SLS and DOSS which were absent in case of NSD and DPM. The internalized nanocrystals subsequently dissolve in the cellular matrix [28] and permeate to the donor compartment.

A similar trend in solubility as that observed in Caco-2 cell studies was observed in the everted gut sac experiments. The solubility difference for NS and NSD w.r.t. DPM was 1.82- and 1.52-fold, respectively. However, it was interesting to note that despite ‘difference in solubility’, both NS and NSD showed similar permeability. This was further investigated by conducting the mucoadhesion study as nanocrystals have been reported to possess enhanced mucoadhesion besides solubility enhancement and internalization. Everted gut sac of a rat represents the intestinal tissue with mucous as well as a serosal layer. NSD was found to show the highest mucoadhesion followed by NS and DPM (Figure 6 and Figure 7). The adhered particles are expected to dissolve and permeate over a period of time.

Table 5 captures various contributors to the experimental differences in the permeability behaviour. *P*_app_ (apical-to-basolateral), *P*_app_ (basolateral-to-apical), solubility, and internalization were better for NS, while mucoadhesion was better in case of NSD.

Nanocrystals represent an attractive approach for an increasing number of poorly water-soluble compounds in the product pipeline. Despite the benefits of increased apparent solubility, dissolution rate and permeability, the nanocrystal technology has some limitations. Physical instability of nanosuspensions, which includes sedimentation, crystal growth (also known as Ostwald ripening), aggregation and solid-state transformation, pose challenge in the top-down approach. Another well-known issue with the nanosuspensions is the possible degradation of drugs during generation of nanocrystals (such as due to milling-induced stresses). One of the main advantages of nanocrystalline solid dispersions over nanosuspensions is their physical stability. The final product is obtained as a solid powder and hence, more stable than nanosuspensions. Therefore, exploring nanocrystalline solid dispersions as an alternative to the nanosuspensions, without compromising the desired performance, is an area of work for future work.

## 5. Conclusions

Two nanocrystal-based formulations, i.e., nanosuspension and NanoCrySP-generated nanocrystalline solid dispersion, were compared for their permeability behaviour, cellular internalization, and mucoadhesion. The interplay of concentration-gradient created by the solubility values and mucoadhesion imparted their permeability behaviour. Everted gut sac experiments enabled an understanding of the role of mucoadhesion in the overall permeability behaviour. Though the particle size obtained in the NanoCrySP-based formulation was higher than the nanosuspension, the permeability of the former was comparable to the latter. This work establishes that, besides solubility differences and cellular internalization, mucoadhesion is one of the major contributors to overall permeability in GIT. Mucoadhesion can maintain the local supersaturation and concentration flux by embedded crystals on the mucosa to facilitate absorption. This makes the nanocrystalline solid dispersions an affordable and quality formulation for the industry.

## Figures and Tables

**Table 1:** The solubility of DPM in different media used during in vitro and ex vivo studies. Values are expressed in µg/mL as mean ± standard deviation.

Time (min)	HBSS pH 6.5 (Apical Medium)			HBSS pH 7.4 (Basolateral Medium)			Medium 199		
	DPM	NS	NSD	DPM	NS	NSD	DPM	NS	NSD
15	4.39 ± 0.06	10.32 ± 0.25	8.78 ± 0.33	7.77 ± 1.11	23.46 ± 1.52	13.24 ± 1.85	5.05 ± 0.11	10.32 ± 0.25	10.41 ± 0.74
30	8.95 ± 0.98	31.40 ± 0.92	20.83 ± 0.95	14.88 ± 0.48	46.62 ± 2.59	22.77 ± 1.37	16.62 ± 2.44	31.40 ± 0.92	24.79 ± 1.86
60	24.03 ± 1.38	47.46 ± 2.88	40.74 ± 4.03	16.99 ± 2.50	57.97 ± 0.11	41.93 ± 0.64	33.65 ± 0.71	47.76 ± 2.88	44.32 ± 2.17
90	36.97 ± 1.82	71.93 ± 2.43	48.50 ± 3.79	31.41 ± 1.56	70.50 ± 1.54	53.89 ± 0.29	35.71 ± 0.15	60.41 ± 0.10	50.09 ± 0.31
120	52.18 ± 0.74	85.61 ± 1.78	75.21 ± 1.40	47.06 ± 8.42	79.36 ± 2.21	63.76 ± 0.28	39.53 ± 2.57	71.93 ± 2.43	59.97 ± 0.31

**Table 2:** Bi-directional *P*_app_ and ER data of the coarse DPM, NS, and NSD samples in Caco-2 cell monolayer. Values are represented as mean ± standard deviation.

Sample	*P*_app_ (Apical-to-Basolateral) (10^−6^ cm/s)	*P*_app_ (Basolateral-to-Apical) (10^−6^ cm/s)	ER
DPM	16.84 ± 0.37	61.01 ± 0.51	3.60 ± 0.28
NS	55.42 ± 1.08	25.87 ± 0.25	0.46 ± 0.23
NSD	33.58 ± 0.91	28.25 ± 0.48	0.84 ± 0.35

**Table 3:** Absorption and desorption of DPM in Caco-2 cells from DPM, NS and NSD samples. Data are expressed as the mean ± standard deviation.

Sample	% DPM Absorbed	% DPM Desorbed
DPM	3.55 ± 1.56	25.58 ± 2.13
NS	12.86 ± 1.01	55.05 ± 3.20
NSD	9.91 ± 1.20	51.72 ± 2.30

**Table 4:** *P*_app_ (jejunum and ileum segments) and intestinal tissue uptake (jejunal segment) values of DPM, NS, and NSD in everted gut sac. Data are expressed as the mean ± standard deviation (*n* = 3).

Sample	*P*_app_ (Jejunum) (10^−7^ cm/s)	*P*_app_ (Ileum) (10^−7^ cm/s)	Intestinal Tissue Uptake (ng/cm^2^)
DPM	29.64 ± 1.42	8.36 ± 0.52	336 ± 29
NS	54.34 ± 2.91	35.34 ± 2.51	707 ± 31
NSD	51.68 ± 1.58	40.73 ± 2.70	975 ± 53

**Table 5:** Comparison of performance of DPM, NS and NSD w.r.t. various contributors studied in the present work.

Contributors	DPM *	NS	NSD
Solubility (in HBSS pH 6.5 after 120 min)	1.00	1.64	1.44
Solubility (in medium 199 after 120 min)	1.00	1.82	1.52
*P*_app_ (apical-to-basolateral)	1.00	3.30	2.35
*P*_app_ (basolateral-to-apical)	1.00	0.54	0.87
*P*_app_ (mucosa-to-serosa)	1.00	1.83	1.74
Internalization (absorption-desorption study)	1.00	3.62	2.79
Mucoadhesion	1.00	1.42	2.47

* Contribution of DPM has been assumed to be 1 for comparison of the performance of NS and NSD in various aspects.

## Supplementary Materials

The following are available online at http://www.mdpi.com/1999-4923/10/3/160/s1, Table S1: Particle size analysis for stability evaluation of NS and NSD for 30 days.
