# Supplementary material for: Permeability Behavior of Nanocrystalline Solid Dispersion of Dipyridamole Generated Using NanoCrySP Technology
Source: Pharmaceutics. 2018 Sep 17;10(3):160. doi: 10.3390/pharmaceutics10030160 (PMC6161304; doi:10.3390/pharmaceutics10030160)
Supplement: Supplementary file 1 [file pharmaceutics-10-00160-s001.pdf]

pharmaceutics

Supplementary Materials: Permeability Behavior of Nanocrystalline Solid Dispersion of Dipyridamole Generated using NanoCrySP Technology

Ashish Girdhar, Poonam Singh Thakur, Sneha Sheokand and Arvind K. Bansal

Table S1. Particle size analysis for stability evaluation of NS and NSD for 30 days.

Time	NS			NSD		
(day)	Z_{avg} (nm)	D_{90} (nm)	PDI	Z_{avg} (nm)	D_{90} (nm)	PDI
0	373.4 ± 38.2	260.3 ± 34.9	0.1 ± 0.0	1131.0 ± 79.3	720.0 ± 56.9	0.5 ± 0.2
1	395.0 ± 25.6	337.5 ± 22.1	0.1 ± 0.0	1072.1 ± 51.6	663.0 ± 42.6	0.5 ± 0.1
3	295.5 ± 24.9	261.5 ± 37.7	0.1 ± 0.0	1096.7 ± 87.0	760.1 ± 34.3	0.5 ± 0.1
9	355.0 ± 13.0	306.6 ± 40.4	0.1 ± 0.1	1141.4 ± 63.3	664.3 ± 62.9	0.4 ± 0.1
15	342.7 ± 24.2	260.8 ± 29.4	0.1 ± 0.0	983.0 ± 76.3	563.1 ± 98.9	0.5 ± 0.1
30	343.6 ± 32.6	275.3 ± 35.5	0.2 ± 0.0	1115.1 ± 76.0	721.4 ± 44.4	0.5 ± 0.2
